# Effect of Electrical and Electromechanical Stimulation on PC12 Cell Proliferation and Axon Outgrowth

**DOI:** 10.3389/fbioe.2021.757906

**Published:** 2021-10-21

**Authors:** Kailei Xu, Xixia Liu, Xiaokeng Li, Jun Yin, Peng Wei, Jin Qian, Jie Sun

**Affiliations:** ^1^ Central Laboratory, Ningbo First Hospital, Ningbo, China; ^2^ The State Key Laboratory of Fluid Power and Mechatronic Systems, School of Mechanical Engineering, Zhejiang University, Hangzhou, China; ^3^ Key Laboratory of 3D Printing Process and Equipment of Zhejiang Province, School of Mechanical Engineering, Zhejiang University, Hangzhou, China; ^4^ School of Mechanical Engineering, Guizhou University, Guiyang, China; ^5^ Department of Hand and Foot Microsurgery, Ningbo First Hospital, Ningbo, China; ^6^ Key Laboratory of Soft Machines and Smart Devices of Zhejiang Province, Department of Engineering Mechanics, Zhejiang University, Hangzhou, China; ^7^ Department of Neurosurgery, Ningbo First Hospital, Ningbo, China

**Keywords:** nerve regeneration, electrical field, mechanical stretch, silver nanowires, nerve cells, PDMS

## Abstract

Peripheral nerve injuries have become a common clinical disease with poor prognosis and complicated treatments. The development of tissue engineering pointed a promising direction to produce nerve conduits for nerve regeneration. Electrical and mechanical stimulations have been incorporated with tissue engineering, since such external stimulations could promote nerve cell proliferation, migration and differentiation. However, the combination of electrical and mechanical stimulations (electromechanical stimulation) and its effects on neuron proliferation and axon outgrowth have been rarely investigated. Herein, silver nanowires (AgNWs) embedded polydimethylsiloxane (PDMS) electrodes were developed to study the effects of electromechanical stimulation on rat pheochromocytoma cells (PC12 cells) behaviors. AgNWs/PDMS electrodes demonstrated good biocompatibility and established a stable electric field during mechanical stretching. PC12 cells showed enhanced proliferation rate and axon outgrowth under electrical stimulation alone, and the cell number significantly increased with higher electrical stimulation intensity. The involvement of mechanical stretching in electrical stimulation reduced the cell proliferation rate and axon outgrowth, compared with the case of electrical stimulation alone. Interestingly, the cellular axons outgrowth was found to depend on the stretching direction, where the axons prefer to align perpendicularly to the stretch direction. These results suggested that AgNWs/PDMS electrodes provide an *in vitro* platform to investigate the effects of electromechanical stimulation on nerve cell behaviors and can be potentially used for nerve regeneration in the future.

## Introduction

The nervous system, composed of the central nervous system and the peripheral nervous system, is a vast conductive network that coordinates actions and sensory information of the human body through the transmission of electrical signals to and from different tissues. The central nervous system includes the brain and the spinal cord; while the peripheral nervous system is composed of numerous long fibers or axons, which connect with the central nervous system. Peripheral nervous injuries (PNI) have become a common clinical disease, with millions of cases happening annually around the world, and more than 200,000 repair surgeries are performed in the US every year (Du et al., 2018). The PNI generally causes a poor prognosis due to the complicated treatments and high morbidity. About 60% of PNI patients have poor rehabilitation of exercise abilities, and some of them require long-term care by others, which significantly affects patients’ life quality (Gu et al., 2011; Wieringa et al., 2018). Therefore, it is of great importance to develop effective PNI repair strategies.

Clinical treatments for PNI have not changed much in the past decades, mainly including end-to-end and fascicular suture repair techniques, grafting sensory nerves, and autografts as the gold standard. However, the autografts still have many limitations, like donor repeated operations, nerve unavailability, local tissue adhesion, and size mismatch (Daly et al., 2013; Tang et al., 2013; Jahromi et al., 2019). Recently, the development of tissue engineering has pointed a new direction on PNI treatment, i.e., nerve conduit (Suo et al., 2018; Liu et al., 2021; Wang et al., 2021), which is an artificial hollow tube as an alternative that connects the two ends of the injured nerve to support nerve migration and regeneration.

Successful nerve conduit needs to provide not only physical support for growing neurites (Yin et al., 2017; Zhang et al., 2020), but also biological signals to promote nerve cell migration, proliferation, axon outgrowth, and orientation (Huang and Huang, 2006). These behaviors of nerve cells in tissue engineering could be influenced by many cues of substrate surface topographies, like aligned fibers, roughness, and pores, and external stimulations (Fozdar et al., 2010; Hoffman-Kim et al., 2010; Clarke et al., 2011). Electrical stimulation has been shown to promote neurite outgrowth, axon regeneration, and neural repair *in vitro* and *in vivo* (Kotwal and Schmidt, 2001; Bhang et al., 2012; Du et al., 2016), which might be due to its promotion effect on nerve cell adhesion, growth, migration, and proliferation. Micropatterned silk protein films sputter-coated with metal electrodes have been shown to promote the nerve cell axon outgrowth under electrical stimulation (Hronik-Tupaj et al., 2013). Animal studies also demonstrated that electrical stimulation can accelerate the maturity of regenerated nerves (Lu et al., 2008). Both peripheral nerve fibers and cells are constantly subjected to stretching and compression forces during the movement of the limbs (Phillips et al., 2004); therefore, the PNI repair and nerve cell growth are also affected by mechanical stimulation (Ishibashi et al., 2016; Wu et al., 2019). The adhesion (Love et al., 2017), signaling pathways (Grove and Brophy, 2014), and gene expression (Gupta et al., 2012) of nerve cells have been found to be tightly regulated by mechanical stimulations, including the change of tissue stiffness or shear stress. Although significant progress has been made in the application of electrical stimulation and mechanical stimulations for neural tissue engineering, the combination of these two factors was rarely investigated.

In this study, polydimethylsiloxane (PDMS) was combined with silver nanowires (AgNWs) to prepare AgNWs/PDMS electrodes that were used to study the responses of rats pheochromocytoma (PC12) cells to electrical stimulation as well as electromechanical stimulation. AgNW has been widely used for flexible/wearable electrodes or stretchable applications, due to its excellent electrical conductivity, high mechanical strength and good biocompatibility. The role of AgNWs in this study acted as a conductive electrode material to provide a stable electrical field to study the effect of electrical stimulation on PC12 cellular behaviors. PDMS was a commonly used substrate for implantable electrodes, due to its low water absorption and high flexibility and elasticity (Jeong et al., 2016). Our group has used PDMS as substrate to study the behavior of PC12 cells under mechanical stimulation alone previously (Lin et al., 2020). PC12 cells demonstrated axon orientation perpendicular to the stretch direction, while the cell proliferation was not influenced. The role of PDMS in this study is to prevent the direct contact between PC12 cells and AgNWs, and also provide sufficient mechanical strength and high elasticity to protect the AgNWs from breaking during electromechanical stimulation. The prepared AgNWs/PDMS electrodes were characterized with a scanning electron microscope (SEM) for the microscopic surface structure of the AgNWs layer, and the electromechanical properties of AgNWs/PDMS electrodes were characterized by cyclic voltammetry during the cyclic tensile test using a universal resistance meter. *In vitro* cell culture was performed with PC12 cells on the electrodes for 120 h and analyzed with Calcein-AM for cell viability, fluorescence imaging for cell density counting, cell axon outgrowth and orientation measurement at 24, 72, and 120 h. These results demonstrated that the AgNWs/PDMS electrodes are compatible with nerve cell culture as an integrated electromechanical stimulator for cell proliferation and axon outgrowth.

## Methods

### Materials

PDMS was purchased from Sylgard (United States). AgNWs (diameter 30–50 nm, length 10–30 μm, 10 mg/ ml in DMF) were purchased from ECHUANG (China). Dulbecco’s modified Eagle’s medium (DMEM) was purchased from Hyclone (Logan, UT). Poly-L-lysine (PLL) was purchased from Beyotime (China). Fetal bovine serum (FBS) was purchased from Gibco (Waltham, MA). Penicillin/streptomycin (P/S) was purchased from Invitrogen (Carlsbad, CA, United States). Calcein-AM was purchased from Aladdin (China). The PC12 cell line was purchased from Cell Bank of the Chinese Academy of Science (Shanghai, China, Catalog #: TCR9), which are highly differentiated already. PDMS is a commonly used elastomer due to its good biocompatibility and mechanical properties, and Ag-based materials have inherently high electrical conductivity (Han et al., 2019). PC12 cells are widely used to study neural behavior since they could differentiate into the nerve cells, which have neurite, dendrite, and axon, and form synapses with neighboring cells to build neural networks (Foley et al., 2005).

### Preparation of AgNWs/PDMS Electrode

**FIGURE 1:**
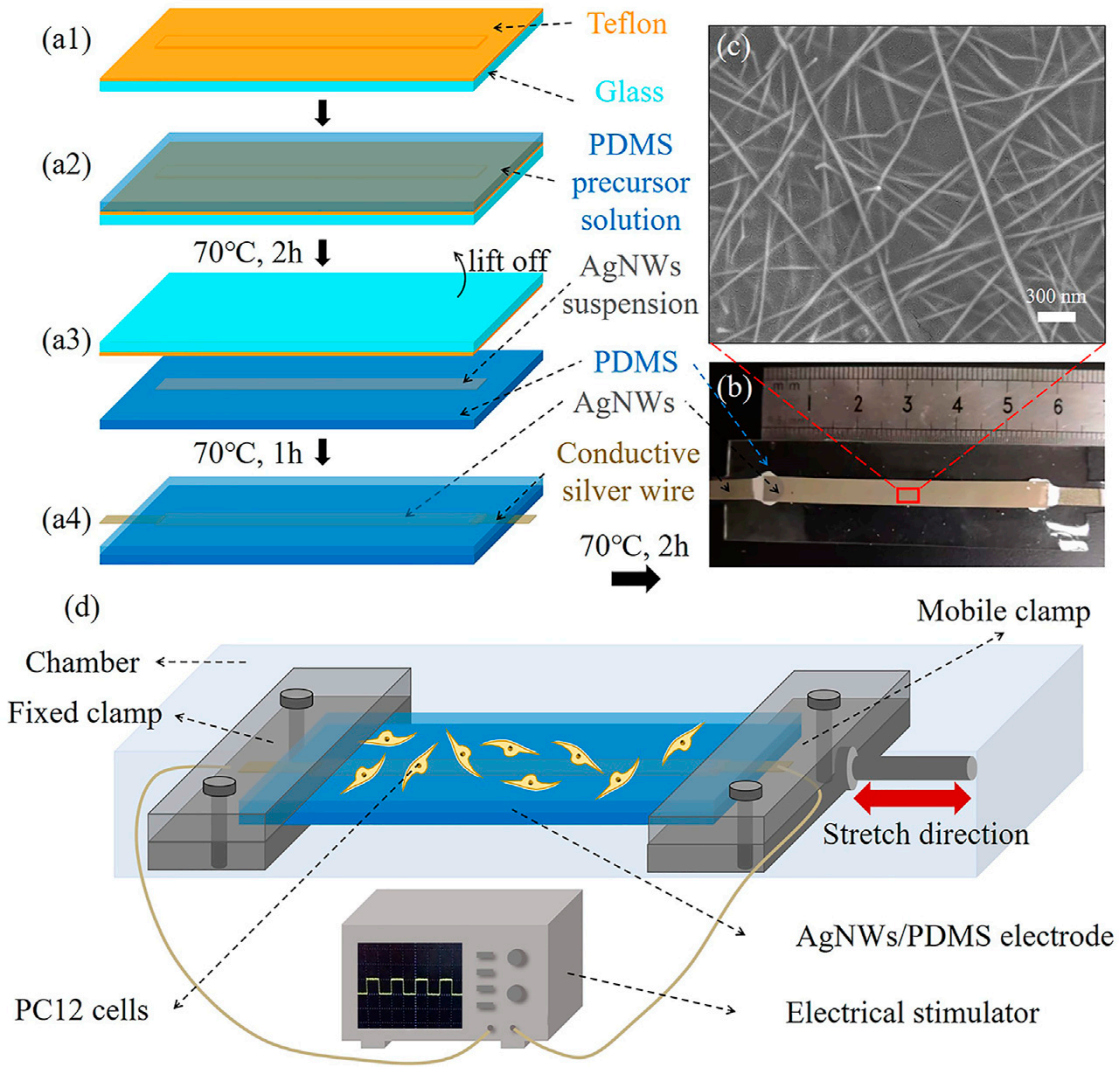
**(A)** Fabrication procedure of the AgNWs/PDMS electrode preparation. **(B)** Photograph of the prepared AgNWs/PDMS electrode. **(C)** SEM image of the surface of the AgNWs layer in the AgNWs/PDMS electrode. **(D)** Schematic illustration of electromechanical stimulation.

The process for the preparation of AgNWs/PDMS electrodes is shown in Figure 1A, including four procedural steps. First, a glass slide (75 mm × 25 mm) was cleaned with 70% ethanol, blow-dried, and attached with a Teflon mold that had a convex part [5 mm × 70 mm × 0.1 mm, (Figure 1A)]. Second, the PDMS precursor solution was prepared by mixing pre-polymer gel and crosslinker at a ratio of 10:1 (w/w), then coated on the Teflon mold to form a thickness of 0.5 mm and solidified at 70°C for 2 h (Figure 1A). Third, the solidified PDMS was demoulded and inverted, filled with 150 μL of AgNWs suspension into the rectangular groove (5 mm × 70 mm × 0.1 mm) that formed by the convex part of the Teflon mold, and dried at 70°C for 1 h to remove DMF (Figure 1A). In particular, the conductive silver wires were connected to the two ends of solidified AgNWs with conductive glue (Figure 1A). Finally, the PDMS substrate containing AgNWs and conductive wires were covered with 0.5 mm-thick PDMS precursor solution and then solidified at 70°C for 2 h to realize the encapsulation and obtain AgNWs/PDMS electrodes (Figure 1B).

### SEM Imaging

The surface morphology of the deposited AgNWs layer was observed by coating with a thin layer of gold using a scanning electron microscope (SU8010, Hitachi, Japan), where two PDMS layers of the electrodes were stripped and the PDMS base layer was removed.

### Electromechanical Properties of the AgNWs/PDMS Electrode

Excellent stretchability and electrical stability in the stretched state are the basis of electrodes for biomedical applications. The electromechanical properties of the prepared AgNWs/PDMS electrodes were characterized by cyclic voltammetry. Briefly, the electrical resistance of the AgNWs/PDMS electrode was recorded under normal and stretch conditions using an electronic extensometer (UTM17290-220, Shenzhen SUNS, China) and a universal resistance meter (KEYSIGHT, 34465A, China). The AgNWs/PDMS electrode was clipped onto the extensometer with an initial effective stretching distance of 30 mm. A periodic tensile test was applied to the sample with a crosshead speed of 1.25 mm/ s. The electrode sample was elongated to reached 30% strain (9 mm) and then relaxed back to 0% strain as one stretching cycle. This was repeated 100 times, and the resistance was measured/recorded by the resistance meter at a frequency of 2 Hz.

### Cell Culture on AgNWs/PDMS Electrode Under Electrical Stimulation

PC12 cells were expanded in the Petri dishes until 70% confluency at 37°C and 5% CO_2_ in a humidified incubator (Thermo Scientific, United States). The fully supplemented media for PC12 cell culture was DMEM with 10% FBS and 1% P/S. The cells were detached from the Petri dish with trypsin-EDTA, centrifuged at 1000 RPM for 5 min, and resuspended in fully supplemented media at a concentration of 8 × 10^4^ cells/mL. The AgNWs/PDMS electrode was coated with PLL and sterilized with 75% ethanol. Then 5 ml of cell suspension was added to the surface of the AgNWs/PDMS electrode and incubated at 37°C and 5% CO_2_ in a humidified incubator. An electrical stimulator (Tektronix, United States) was connected to the AgNWs/PDMS electrode and provided a square wave voltage with different amplitude (60 mV, 120 mV, 240 mV) at a frequency of 20 Hz. Cells were cultured for 120 h and stimulated for 4 h per day, which should not cause bio-demage. Similar electrical stimulation conditions were also performed in other studies. PC12 cells cultured on conducting polymer polypyrrole were stimulated with 720 mV to investigate the extracellular matrix change (Kotwal and Schmidt, 2001). Schwann cells were electrically stimulated under 200 mV continuously for a maximum of 48 h with little necrosis (Du et al., 2016).

### Electromechanical Stimulation on Cell Culture

The AgNWs/PDMS electrode was seeded with 5 ml of cell suspension and incubated for 4 h for cell attachment. Then the sample was transferred to a biological tensile tester (MechanoCulture T6, CellScale, Canada), exposed to cyclic tensile stretching (10% strain at 0.25 Hz) and electrical stimulation with a square wave voltage of 240 mV and 20 Hz. Cell culture without any stimulation was served as a control.

### Fluorescence Stain for Cell Proliferation and Axon Outgrowth Analysis

PC12 cells were stained with Calcein-AM for cell morphology imaging. The electrode seeded with PC12 cells were washed with D-PBS and incubated with 1 μg/ ml Calcein-AM for 30 min at 37°C and 5% CO_2_ in an incubator. The sample was washed with D-PBS three times and imaged with a fluorescence microscope (Ti-S, Nikon, Japan). The axon outgrowth of PC12 cells was characterized by length (L) and the orientation (θ), where L is the end-to-end distance from soma to axon tip and θ is the angle between axon and electric current or stretch direction (Shineh et al., 2019; Krishnamoorthy et al., 2020).

### Statistical Analysis

All quantitative data were analyzed and presented as the mean ± standard deviation and the comparison between two means was analyzed using Tukey’s tests, in which *p* < 0.05 was considered statistical significance. For cell density measurement, more than 10 images were analyzed. For axon orientation and length, the distribution of orientation θ was divided into 12 uniform intervals with a span of 30° (Lin et al., 2020). Each data of axon length and cell orientation was obtained based on the measurements (*n* > 150 cells for each condition) using ImageJ (version 1.8.0, National Institutes of Health, United States).

## Results

### AgNWs/PDMS Electrode Characterization

**FIGURE 2:**
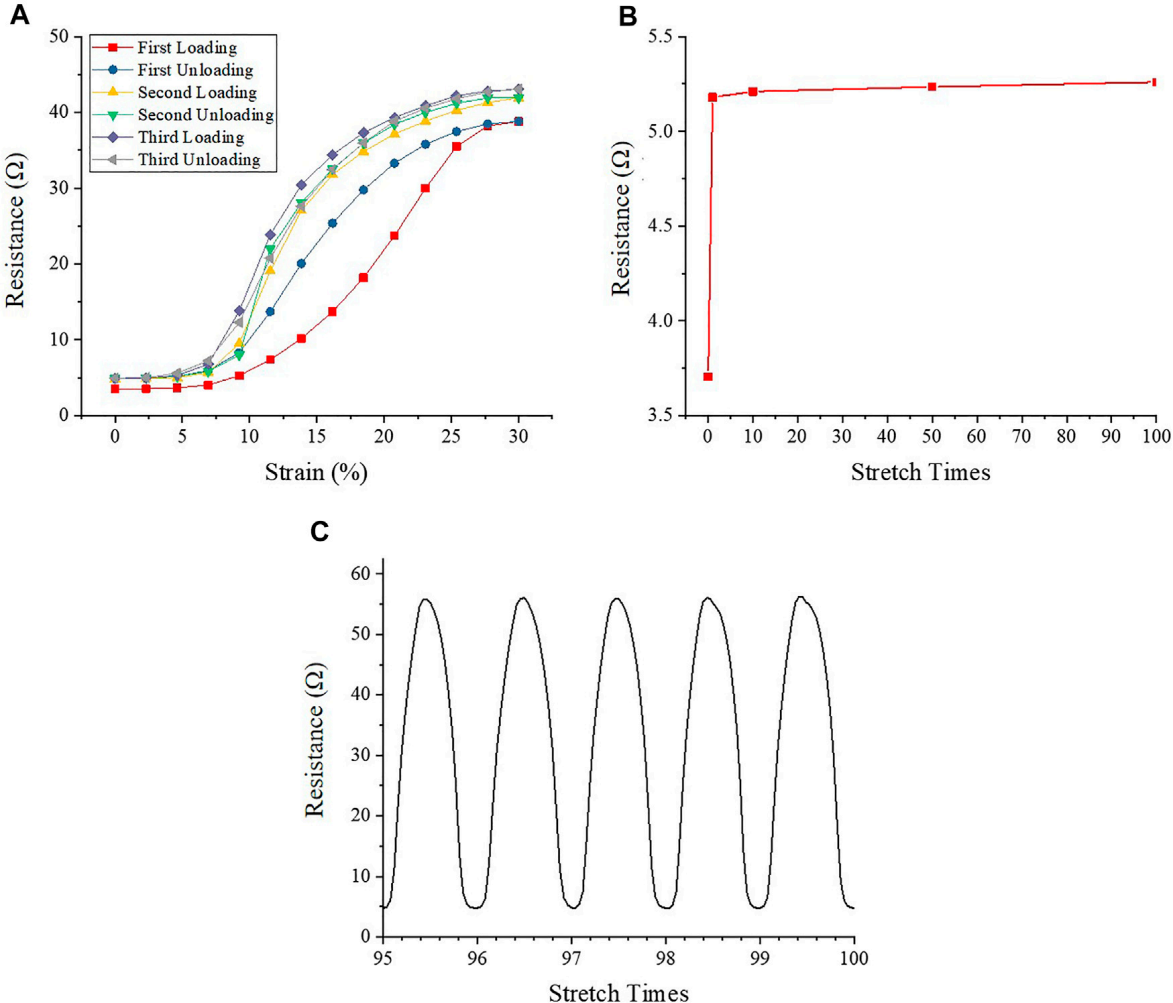
The electrical resistance of AgNWs/PDMS electrode under mechanical loading cycles. **(A)** Electrical resistance during repeated stretch-relaxation cycles (the first to third cycles). **(B)** Electrical resistance of AgNWs/PDMS electrode with no strain after stretch. **(C)** Electrical resistance during repeated stretch-relaxation cycles (the last five cycles).

Representative AgNWs/PDMS electrode with a total length of ∼60 mm is shown in Figure 1B. It is noted that the pattern of electrodes (AgNWs) could be easily modified by changing the shape of the Teflon mold through 3D printing. The SEM image of the AgNWs surface after pilling off the PDMS substrate was shown in Figure 2C. Originally, the AgNW nanofibers were brittle and easy to fracture, however, the AgNW nanofibers in the prepared electrodes formed an interconnected network with PDMS penetration (Figure 2C) due to its flowability (viscosity: 5,467 ± 370 mPas) and low surface energy of the encapsulation layer PDMS precursor solution, resulting in an excellent elasticity and tensile electrical stability of the electrodes.

Figure 2 showed the resistance variation of the electrodes during the cyclic tensile stretching process. The initial resistance of the electrodes was only 3.71 Ω, indicating that AgNWs/PDMS electrodes had good electrical conductivity. During the first stretch-relaxation cycle, the resistance increases to 38.81 Ω, which is almost 10 times higher than the initial value. After the strain was fully released, the resistance recovered back to 5.18 Ω, which was still 40% higher than the initial resistance. The resistance showed a major hysteresis during the first stretch-relaxation cycle, but essentially no hysteresis was found for the subsequent cycles (Figure 2A), indicating that the plastic deformation of the AgNWs during multiple loading cycles was mainly caused by the first stretch-relaxation cycle. The resistance increased from 5.18 to 41.94 Ω during the second stretch-relaxation cycle, which was 8.1% higher than the first cycle, and it returned to 5.18 Ω after the stretch was released. In the third stretch-relaxation cycle, the electrode resistance increased from 5.18 to 43.06 Ω. The resistance value increased by 2.7% compared to the second cycle, indicating that the maximum resistance of the electrode increased with the increase of stretch times during the first three cycles. Due to the variation of electrode resistance, the equivalent current also varies from 16 to 64 mA. While the PC12 cells were cultured in an electrical field rather than in contact with AgNWs directly, the cell viability could be ensured.

To explore the changes of electrode resistance after multiple stretches, a hundred stretch-relaxation cycles test was applied to the electrode samples. Figure 2B showed the change of resistance at the relaxation point, and it could be found that after increasing from 3.71 to 5.18 Ω during the first three cycles, the resistance of the electrode samples was almost unchanged during the subsequent cycles. The resistance change in the last five stress-relaxation cycles was similar (Figure 2C), and the average change of the maximum resistance was less than 0.05 Ω, which is negligible.

### Effects of Electrical Stimulation on PC12 Cell Growth

**FIGURE 3:**
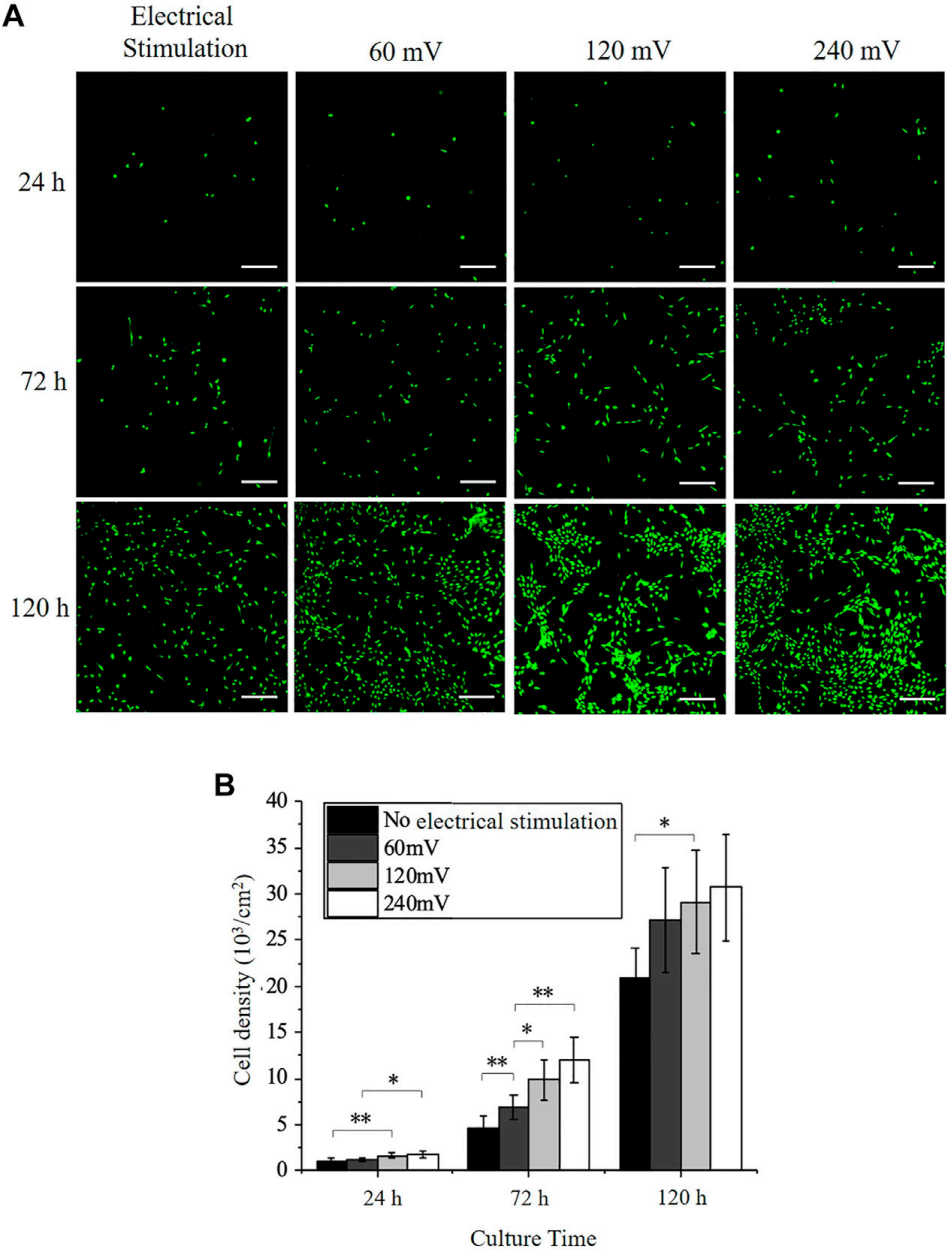
PC12 Immunofluorescence staining and cell proliferation under electrical stimulation. **(A)** Immunofluorescence images of PC12 cells cultured without or with electrical stimulation. Scale bar: 200 μm. **(B)** Cell densities of PC12 cultured on the electrodes. The significant difference between all the groups is indicated (∗, *p* < 0.05; ∗∗, *p* < 0.01; *n* > 10 images).

The calcein AM stained PC12 cells were imaged at 24, 72, and 120 h (Figure 3A). More cells were observed on the electrode surface under electrical stimulation compared with the control group. This was further verified with the cell number measurement using Image J (version 1.8.0, National Institutes of Health, United States). As shown in Figure 3B, the cell number on the control group without electrical stimulation was (1.07 ± 0.32) × 10^3^ cells/cm^2^, (4.60 ± 1.25) × 10^3^ cells/cm^2^, and (20.89 ± 3.16) × 10^3^ cells/cm^2^ at 24, 72, and 120 h, respectively. The cell number increased 4.3 times after 72 h and 4.5 times after 120 h, while cells were still not able to cover the whole sample area (Figure 3A). The cell number increased significantly with the intensity of electrical stimulation at 24 and 72 h (Figure 3B). Under 60 mV electrical stimulation, the cell number was (1.21 ± 0.22) × 10^3^ cells/cm^2^, (6.86 ± 1.26) × 10^3^ cells/cm^2^ and (27.14 ± 5.72) × 10^3^ cells/cm^2^ at 24, 72, and 120 h, respectively. Under 120 mV electrical stimulation, the cell number was (1.65 ± 0.26) × 10^3^ cells/cm^2^, (9.84 ± 2.22) × 10^3^ cells/cm^2^ and (29.07 ± 5.58) × 103 cells/cm^2^ at 24, 72, and 120 h, respectively. Under 240 mV electrical stimulation, the cell number was (1.97 ± 0.33) × 10^3^ cells/cm^2^, (11.96 ± 2.44) × 10^3^ cells/cm^2^ and (30.68 ± 5.71) × 10^3^ cells/cm^2^ at 24, 72, and 120 h, respectively. The cell number reached a similar level for all electrical stimulation samples at 120 h and cover the whole cell culture space. The cell number for the 240 mV group increased 6.8 times, while the 60 and 120 mV groups increased 5.6 times and 6 times, respectively, indicating that electrical stimulation can effectively promote the proliferation of PC12 cells, and this effect became more pronounced as the intensity of electrical stimulation increased.

### Effects of Electrical Stimulation on PC12 Cell Axon Outgrowth

**FIGURE 4:**
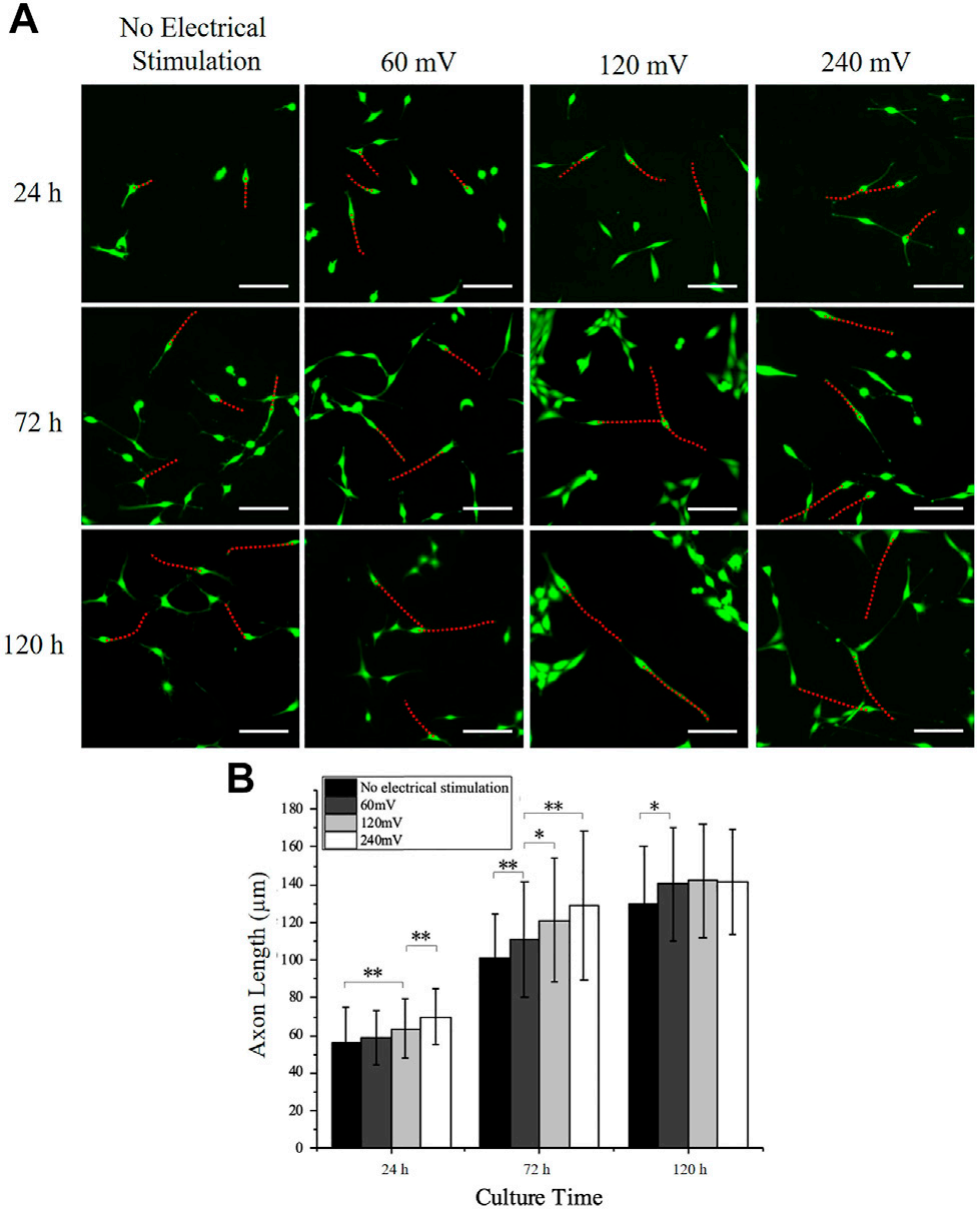
PC12 cell axon outgrowth measurement under electrical stimulation. **(A)** Representative PC12 cells immunofluorescence images for all electrical stimulation groups. Scale bar: 100 μm. **(B)** Axon length measurement of PC12 cells cultured on the electrodes. The significant difference between groups is indicated (∗, *p* < 0.05; ∗∗, *p* < 0.01; *n* > 150 cells).

The PC12 cell axon outgrowth was also analyzed and indicated with red dotted lines (Figure 4A). The length of the axon of the PC12 cells increased after from 24 to 120 h in all groups. The axon length of PC12 cells on control group at 24, 72, and 120 h were 55.81 ± 18.79 μm, 100.96 ± 23.77 μm, and 129.67 ± 30.46 μm, respectively. The axon length increased by 45 μm from 24 to 72 h and only increased by 29 μm from 72 to 120 h, indicating that the outgrowth rate of the axon decreased with time in the control group. The axon length of PC12 cells under 60 mV at 24, 72, and 120 h was 58.98 ± 14.21 μm, 111.15 ± 30.54 μm, and 140.58 ± 30.15 μm, respectively; the axon length of PC12 cells under 120 mV at 24, 72, and 120 h was 63.55 ± 15.76 μm, 121.13 ± 32.72 μm, and 142.04 ± 30.38 μm, respectively; and the axon length of PC12 cells under 240 mV at 24, 72, and 120 h was 70.01 ± 14.77 μm, 128.53 ± 29.6 μm, and 141.18 ± 27.86 μm, respectively. The axon outgrowth was slightly increased as the increases of electrical stimulation intensity at 24 h, although significant differences were only found between the 120 mV and the control group, and the 240 mV and the 120 mV group. The same phenomenon was also observed at 72 h with more significant differences observed, between the 60 mV and the control group, the 120 mV and the 60 mV, and the 240 mV and the 60 mV. All the three electrical stimulation groups showed similar axon outgrowth after 120 h, but slightly higher than the control group, indicating that electrical stimulation could promote axon outgrowth.

### Effects of the Electromechanical Stimulation on PC12 Cell Outgrowth

**FIGURE 5:**
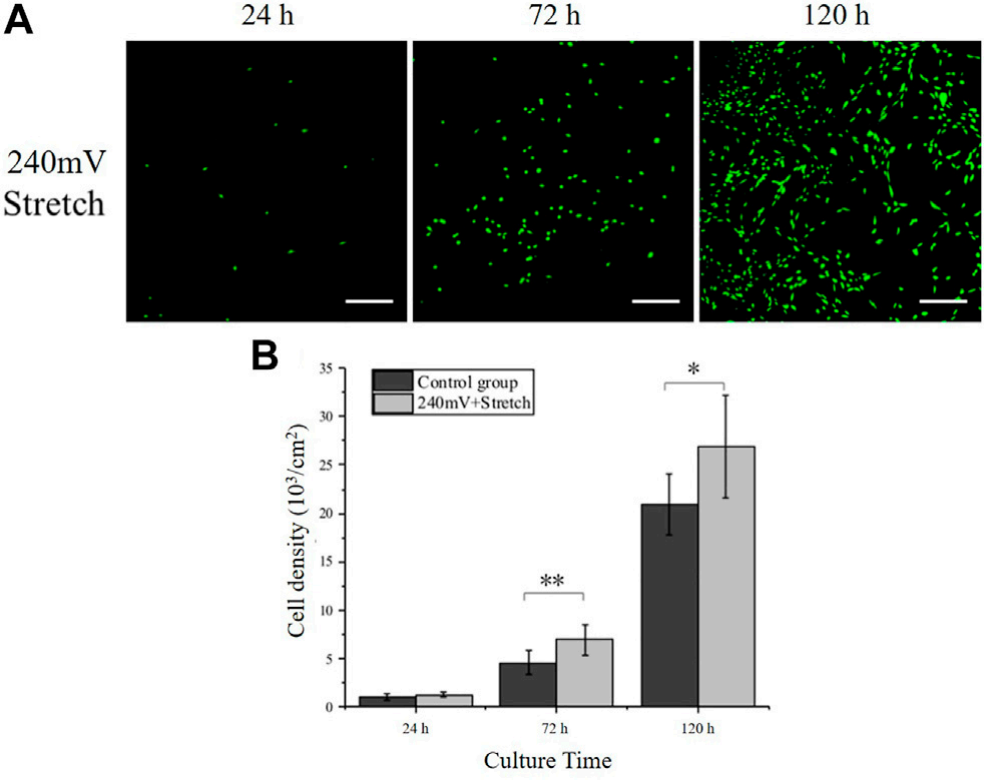
PC12 cell proliferation under electromechanical stimulation. **(A)** Immunofluorescence images of PC12 cells cultured under electromechanical stimulation. electrical stimulation: 240 mV, 20 Hz; Stretch: 10%, 0.25 Hz. Scale bar: 100 μm. **(B)** Cell densities measurement. The significant difference between groups is indicated (∗, *p* < 0.05; ∗∗, *p* < 0.01; *n* > 10 images).

PC12 cells were cultured on the electrodes under electromechanical stimulation to analyze the effects on the PC12 cell proliferation and axon outgrowth. Based on the electrical stimulation alone results (*Effects of Electrical Stimulation on PC12 Cell Growth* to *Effects of Electrical Stimulation on PC12 Cell Axon Outgrowth* *)*, the electrical stimulation intensity of 240 mV in combination with the mechanical stretching stimulation with a tensile strain of 10% and a frequency of 0.25 Hz was used as the electromechanical stimulation parameters. The PC12 cell densities (Figure 5) under electromechanical stimulation at 24, 72, and 120 h were (1.23 ± 0.24) ×10^3^ cells/cm^2^, (6.94 ± 1.59) ×10^3^ cells/cm^2^ and (26.88 ± 5.36) ×10^3^ cells/cm^2^, respectively. Compare with the control group (without any stimulation), PC12 cells under electromechanical stimulation had significantly larger cell numbers at 72 and 120 h, indicating that the electromechanical stimulation can also promote cell proliferation. The PC12 cell densities under electromechanical stimulation at all time points were similar to the 60 mV electrical stimulation group, while smaller than the 120 mV electrical stimulation group (Figure 3). This may be attributed to the periodic change of the electrode resistance during stretching, resulting in larger equivalent resistance of the electrodes (Figure 2), and therefore larger voltage was needed to achieve the same effect as the 240 mV samples.

**FIGURE 6:**
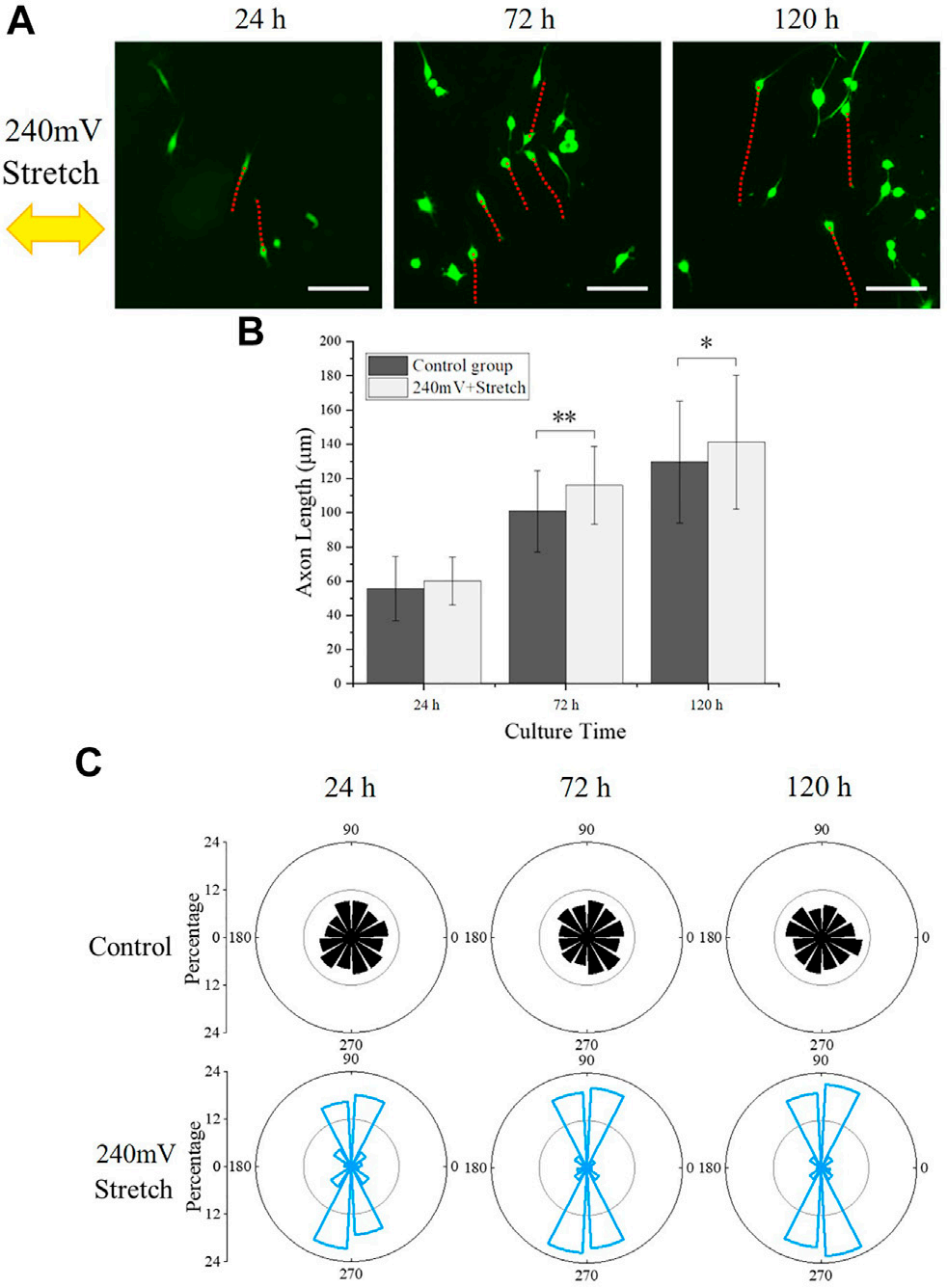
PC12 cell axon orientation analysis. **(A)** PC12 cells fluorescence images under electromechanical stimulation, with red dotted lines indicating the axon outgrowth and the yellow arrow representing the stretching direction. Scale bar: 100 μm. **(B)** Statistical results of axon length. The significant difference between groups is indicated (∗, *p* < 0.05; ∗∗, *p* < 0.01; *n* > 150 cells). **(C)** Statistical angular distribution of axon orientation based on the fluorescence images.

The axon growth of the PC12 cells under the electromechanical stimulation was also analyzed (Figures 6A,B). The axon outgrowth of PC12 cells with electromechanical stimulation at 24, 72, and 120 h were 60.12 ± 13.84 μm, 116.01 ± 22.55 μm, and 141.29 ± 39.15 μm, respectively, which were significantly longer than the corresponding values of the control group. PC12 cells under electromechanical stimulation showed similar axon length as the 60 mV electrical stimulation group but shorter than the 120 mV electrical stimulation group, similar to the phenomenon observed for cell proliferation resulting from the larger equivalent resistance during stretching. The axon length reached 141.29 ± 39.15 μm after 120 h of culture, which was similar to the electrical stimulation alone group that the PC12 cells reached their maximum axon length.

The angle between the axon and stretch direction (θ) was further analyzed (Figure 6C). Interestingly, the axon of PC12 cells under electromechanical stimulation demonstrated a stretch direction-dependent growth, which was not observed in the control group and electrical stimulation alone groups. The percentages of cells that aligned perpendicularly (60° ≤ θ ≤ 120° and 240° ≤ θ ≤ 300°) to the stretching direction were 65.45, 79.72, 84.59% at 24, 72, and 120 h, respectively, indicating that the stretching stimulation influenced the preferred direction of the PC12 cell axon outgrowth.

## Discussion

In this study, AgNWs/PDMS electrode was developed to investigate the effects of electromechanical stimulation on PC12 cell proliferation and axon outgrowth. Although Ag-based materials have inherently high electrical conductivity, their application as biomaterial was limited due to the possible releasement of toxic Ag oxidation and Ag ions (Hirai et al., 2016). In the present work, AgNWs were encapsulated into the PDMS model which prevented the release of toxic Ag ions and improved the electrode biocompatibility, while keeping the benefit of high electrical conductivity and establishment of a stable electric field. The use of the PDMS model not only prevented the leak of toxic byproducts but also provided sufficient mechanical strength and high elasticity to protect the AgNWs from breaking during stretching (Figure 2), which provided a promising platform to investigate the effects of electromechanical stimulation on PC12 cell proliferation and axon outgrowth.

The preceding results have clearly shown that electrical stimulation promotes the PC12 cell proliferation and axon outgrowth on AgNWs/PDMS electrodes. Similar results were found in previous studies: PC12 cells cultured on Polypyrrole-coated silk fibroin nanofibers (Sun et al., 2016) and P19 neurons (Hronik-Tupaj et al., 2013) cultured on silk film showed significantly higher cell proliferation rate and axon extension under electrical stimulation. Such phenomena could be explained by the effect of electrical stimulation on the gene expression and protein synthesis that regulate cell proliferation and axon outgrowth (Kimura et al., 1998). The myelin gene expression and neurotrophin secretion have been enhanced in nerve cells under electrical stimulation, which could lead to a longer axon outgrowth and a higher proliferation rate (Wu et al., 2016). Electrical stimulation was also found to upregulate the secretion of ciliary neurotrophic factor, glial cell-derived neurotrophic factor and fibroblast growth factor I, which could enhance the axon growth (Du et al., 2016). PC12 cellular membrane proteins, like F-actin and stress fibers, are critical factors that control the axon outgrowth, which are exposed to the mechanical stimulations first. When stretching amplitude and frequency are sufficiently large, cellular stress fibers are not able to maintain the axon orientations parallel to the cyclic stretch direction, and PC12 cells are forced to rotate to directions that are nearly perpendicular to the stretch direction (Lin et al., 2020). Another hypothesis is that cells will overexpress specific enzymes, like lysozyme, under mechanical stimulation, which could change the cellular protein expression, morphology, axon growth and direction (Kawasaki et al., 2009a; Kawasaki et al., 2009b; Ito et al., 2011). We plan to investigate the cell behavior mechanisms in our future studies. The cell number and axon outgrowth became similar for all groups after 120 h, which could be caused by the limitation of cell culture space. When PC12 cells overgrow and axon interaction happens between cells, contact inhibition was triggered. Then, contact inhibition could cease the cell proliferation, division, and axon growth, and finally stop the cell from continuing growth.

To further reveal the effects of electromechanical stimulation on PC12 cell behavior, 240 mV and 10% stretch strain at 0.25 Hz was applied. PC12 cells demonstrated enhanced proliferation and axon outgrowth, in comparison with the control group (Figures 5, 6). However, both cell number and axon outgrowth at all time points were similar as 60 mV electrical stimulation sample, while smaller than 120 mV electrical stimulation sample. This could be due to the periodic change of electrode resistance during stretching, resulting in larger equivalent resistance of the electrode compared with the electrical stimulation alone group. Therefore, a larger voltage could be needed to overcome the electrical resistance change during the stretching and achieve a similar cell proliferation rate. Interestingly, the axon orientation of the PC12 cell was found to be more perpendicular to the stretch direction with electric stimulation and stretch. Embryonic rat cortical neurons obtained from pregnant rats cultured on PDMS also showed similar results, where the direction of neuronal outgrowth was perpendicular to the strain direction (Abraham et al., 2019). This perpendicular relation between axon outgrowth and stretch direction resembled the cyclic strain-induced actin reorientation, which could relate to the function of Src-family kinase activity that controls the cellular reorientation (Niediek et al., 2012). Overall, it was found that electric stimulation alone could enhance the PC12 cell proliferation and axon outgrowth on AgNWs/PDMS electrodes, while the electromechanical stimulation could offset the enhancement and direct the PC12 cell axon orientation.

## Conclusion

In this work, PC12 cells were cultured on the AgNWs/PDMS electrodes to study the effects of electromechanical stimulation on cellular proliferation and axon outgrowth. The AgNWs/PDMS electrodes were developed and characterized to exhibit stable electrical resistance and good elasticity for stretching; therefore, an integrated electromechanical stimulation cell culture platform was established. The *in vitro* cell culture demonstrated that the prepared AgNWs/PDMS electrodes had good biocompatibility and could successfully support the adhesion and proliferation of the PC12 cells. The electric stimulation alone was found to enhance the proliferation rate and axon outgrowth of PC12 cells, and the cell number was increased with the increase of the electric stimulation intensity. However, the cell proliferation rate and axon outgrowth was dropped under electromechanical stimulation compared with the electric stimulation alone groups, while the axon orientation could be directed by the stretching direction. These results suggested that the AgNWs/PDMS electrodes provide a promising *in vitro* platform to investigate the sensing mechanisms of nerve cell behaviors to electromechanical cues, and can be potentially used as nerve conduit materials in the future.

## Data Availability

The original contributions presented in the study are included in the article/Supplementary Material, further inquiries can be directed to the corresponding authors.
